# Understanding how closed-loop stimulation pacing suppresses cardioinhibition in vasovagal syncope

**DOI:** 10.1093/europace/euae046

**Published:** 2024-02-10

**Authors:** J Gert van Dijk

**Affiliations:** Department of Neurology, Leiden University Medical Centre, Albinusdreef 2, 2333 ZA Leiden, The Netherlands

**This editorial refers to ‘Temporal relationship between hemodynamic changes and activation of Closed Loop Stimulation during tilt-induced vasovagal syncope’, by V. Russo *et al*., https://doi.org/10.1093/europace/euae045.**

After years of disappointing results, reports of a new successful pacing method in vasovagal syncope (VVS) were very welcome.^1,2^ The new method used a ‘closed-loop stimulation (CLS) rate-adaptive mode’. The paper by Russo *et al*.^3^ in this issue of *Europace* sheds light on how CLS pacing prevents syncope. As the authors stated, the CLS method was initially developed for a different purpose, not specifically for VVS. Apparently, measuring intracardiac impedance is a key feature in this regard. Russo *et al*. subjected 20 people with a CLS pacemaker to a tilt test to evoke VVS and measured haemodynamics to see what happened, with great success. Of note, the authors used simultaneous video to document syncope clinically, illustrating one of the advantages of adding video recording to tilt testing.^4^

The study by Russo *et al*. prompts various considerations.

## Abolishing cardioinhibition

A crucial contributing factor to the success appears to be that CLS pacing started well before heart rate (HR) and blood pressure (BP) plummeted, as they ultimately nearly always do in VVS.^5,6^ Early attempts at pacing for VVS in contrast aimed to prevent extreme bradycardia and asystole, but by the time asystole appears, BP may well be so low that syncope can hardly be prevented anymore. In fact, asystole during tilt-evoked VVS started *after* patients had already lost consciousness in about one-third of cases.^7^ As such ‘late asystole’ occurred more often in older than in younger patients with VVS,^8^ preventing syncope by preventing asystole was most likely successful in younger patients in whom pacing was contra-indicated anyway. In summary, attempts aimed at the prevention of extreme bradycardia only through pacing had a considerable chance of failure.

However, cardioinhibition (CI), or the vagal decrease of HR in reflex syncope, does not merely consist of extreme bradycardia.^5^ Orthostatic VVS, as observed during tilt tests, starts with excessive venous pooling, apparent as a decrease in stroke volume (SV). While this is normally prevented, for unknown reasons it sometimes is not, although there then is a partial correction effort in the form of an HR increase. If the VVS process continues, the corrective increase of HR suddenly stops, and HR starts to decrease.^5^ The HR turning point marking CI onset is well recognizable during a tilt test. From then on, HR decreases almost linearly, with different speeds in different individuals.^5,6^ In a study of 163 cases of tilt-induced VVS, 149 had easily recognizable CI.^5^ Median HR at the start of CI was at the height of its corrective increase at 98 bpm. Cardioinhibition initially merely represented a weakening of this corrective effort, but even so, CI strongly accelerated the ongoing BP decrease. The realization that CI had a profound effect on BP over the entire CI duration suggested that keeping HR at a high corrective level would slow the BP decrease and give patients time to prevent syncope, e.g. by sitting down.^5,8,9^ It seems that CLS pacing does just that: Russo *et al*. reported that CLS pacing started when HR was around 88 bpm, after which HR increased to 105 bpm and later to 95 bpm. Pacing started at a median of 1.7 min before syncope, which supports the idea that CLS prevented CI altogether: in the earlier-mentioned VVS study,^5^ CI started a median of 58 s before syncope.

## A thought experiment

**Figure 1:**
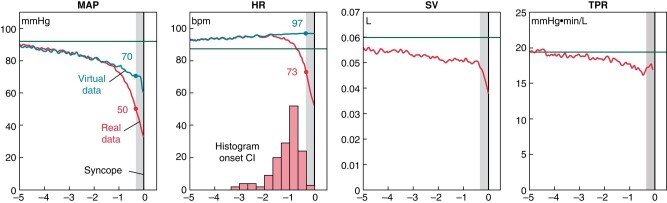
What if CI does not occur? This figure represents a thought experiment, using data of 149 patients with cardioinhibition (CI) and complete syncope.^5^ The time of onset of CI of individual patients was known. The logratio heart rate (HR) value was noted just before the onset of CI and copied to all points later than CI onset, creating a ‘virtual HR’ record. As the logratio value of mean arterial pressure (MAP) for each point in time is the sum of logratio values of HR, stroke volume (SV), and total peripheral resistance (TPR), a ‘virtual MAP’ was formed by adding the logratio values of virtual HR to the unaltered logratio values of SV and TPR. Further calculations reversed the logratio procedure, resulting in ratios and values in original measurement units, shown above. Red lines (bottom ones) show mean original data and blue lines (top ones) show mean virtual HR and MAP data, representing the situation in which CI did not occur. The main result is that without CI, MAP decreased much slower than originally. In reality, SV and TPR underwent large changes in the 20 s before syncope, indicating by a grey zone.^5^ To illustrate the effects of absent CI, values of MAP and HR are indicated for a time point 20 s before syncope in the original data: without CI, HR was 24 bpm higher and MAP 20 mmHg higher than with CI. The time axis represents minutes before syncope. Green horizontal lines represent mean values of the baseline period, just after tilt was completed. At the start of the graph, 5 min before syncope, MAP and SV had already decreased, HR had increased, while TPR was still stable. The histogram in the HR panel represents the time of onset of CI, with a median value of 58 s before syncope.

How much does CI contribute to the decrease of BP in VVS? That question was answered using the ‘logratio analysis’, allowing the contributions of HR, SV, and total peripheral resistance (TPR) to be quantitatively compared.^5^ At syncope, low HR contributed as much to low BP as low SV did, although CI took only 1 min what the decrease of SV had taken up to 10 min.^5^ The logratio method allowed a thought experiment to simulate the complete absence of CI, using the data of 149 patients with CI and syncope (*Figure 1*).

The results revealed that abolishing CI created a progressively larger gap between the real ever faster decrease of mean arterial pressure (MAP) and the slow virtual decrease of MAP without CI. At 20 s before syncope, virtual MAP was 70 mmHg instead of 50 mmHg. This difference is about as much as the upright pressure difference between the heart and the brain, meaning that, as far as brain perfusion is concerned, the difference was as large as between lying and standing.

## How does closed-loop stimulation pacing work in daily life?

Note that the thought experiment slowed the fall of BP but did not stop it. Blood pressure kept decreasing due to a continuing fall of SV (venous vasodepression) and a moderate decrease of TPR (arterial vasodepression).^5,8^ This continuing decrease of BP was noticeably also present in the study by Russo *et al*., in which syncope eventually occurred in most patients.

The fact that CLS did not prevent syncope during a tilt test and simply cannot prevent vasodepression raises the question how well CLS pacing can work in daily life. The slowed BP decrease should provide patients with more time to prevent syncope. If that is the explanation of the pacing success, patients should still experience presyncope from time to time. Unfortunately, the paper did not mention whether or not this was the case, merely that they did not suffer syncope. If patients indeed used presyncope to prevent syncope, then the effect of CLS may be wholly due to diminishing CI. If, however, patients never experienced presyncope, additional factors must explain why syncope occurred during the tilt test but never in daily life. Putative explanations are the use of nitroglycerine during the test or a placebo effect.

Another matter that deserves interest is which value of HR is optimal for CLS pacing. Cardioinhibition in VVS occurs while SV is steadily decreasing. The obvious way to maintain cardiac output when SV is low is to increase HR, but that is not straightforward. Increasing HR shortens cardiac filling time, which by itself may reduce SV. At a certain point, increasing HR reduces SV so much that their product, cardiac output, will decrease. The HR at which cardiac output is maximal is lower when venous return and SV are low,^10^ which holds during VVS. Such considerations suggest that measuring the balance between cardiac output, HR and SV might help to optimize and adapt HR while SV is shifting. Interestingly, the Task Force device used to acquire haemodynamic measurements by Russo *et al*. yields estimates of these parameters, but these are not mentioned in the study.

## Cardioinhibition and vasodepression

The study by Russo *et al*. makes it clear that pacing for VVS is at an interesting crossroads, now that CLS pacing can subtly counter CI. However, vasodepression will still be present, suggesting that measuring the balance between CI and vasodepression might help select patients for pacing. The best VVS candidates are likely to be those in whom CI contributes much more than vasodepression to a BP decrease. Both vasodepression and CI become stronger over time in tilt-evoked VVS, but CI gains strength much quicker. The basic level of CI depends on age^8^ and probably on various other factors. The logratio method allows the contributions of CI, venous and arterial vasodepression to be measured with relative ease. The method can also be used retrospectively, to try to understand why pacing was successful in some patients but not in others. As long as the pathophysiology of VVS has aspects of a black box, as it does, we need pathophysiological studies such as the study by Russo *et al*. to try to open that box, using all the tools available.

## Data Availability

Data are available on reasonable request.
